# Rational Design of Amphiphilic Diblock Copolymer/MWCNT Surface Modifiers and Their Application for Direct Electrochemical Sensing of DNA

**DOI:** 10.3390/polym12071514

**Published:** 2020-07-08

**Authors:** Larisa V. Sigolaeva, Tatiana V. Bulko, Apollinariya Yu. Konyakhina, Alexey V. Kuzikov, Rami A. Masamrekh, Johannes B. Max, Moritz Köhler, Felix H. Schacher, Dmitry V. Pergushov, Victoria V. Shumyantseva

**Affiliations:** 1 Department of Chemistry, M.V. Lomonosov Moscow State University, Leninskie Gory 1/3, 119991 Moscow, Russia; tanyabulko@mail.ru (T.V.B.); konyakhina@list.ru (A.Y.K.); alexeykuzikov@gmail.com (A.V.K.); rami.masamreh@yandex.ru (R.A.M.); pergush@belozersky.msu.ru (D.V.P.); Viktoria.Shumyantseva@ibmc.msk.ru (V.V.S.); 2 V.N. Orekhovich Institute of Biomedical Chemistry, 119121 Moscow, Russia; 3 Pirogov Russian National Research Medical University, 117997 Moscow, Russia; 4 Institute of Organic Chemistry and Macromolecular Chemistry (IOMC), Friedrich-Schiller-University Jena, D-07743 Jena, Germany; johannes.max@uni-jena.de (J.B.M.); moritz.koehler@uni-jena.de (M.K.); felix.shacher@uni-jena.de (F.H.S.); 5 Jena Center for Soft Matter (JCSM), Friedrich-Schiller-University Jena, D-07743 Jena, Germany; 6 Center for Energy and Environmental Chemistry (CEEC), Friedrich-Schiller-University Jena, D-07743 Jena, Germany

**Keywords:** MWCNT dispersion, amphiphilic ionic diblock copolymer, dsDNA, leukocyte DNA, adenine, guanine, DNA electroanalysis, dsDNA direct electrochemical oxidation

## Abstract

We demonstrate the application of amphiphilic ionic poly(*n*-butylmethacrylate)-*block*- poly(2-(dimethylamino)ethyl methacrylate) diblock copolymers (P*n*BMA_40_-*b*-PDMAEMA_40_, P*n*BMA_40_-*b*-PDMAEMA_120_, P*n*BMA_70_-*b*-PDMAEMA_120_) for dispersing multiwalled carbon nanotubes (MWCNTs) in aqueous media, a subsequent efficient surface modification of screen-printed electrodes (SPEs), and the application of the modified SPEs for DNA electrochemistry. Stable and fine aqueous dispersions of MWCNTs were obtained with P*n*BMA_x_-*b*-PDMAEMA_y_ diblock copolymers, regardless of the structure of the copolymer and the amount of MWCNTs in the dispersions. The effect of the diblock copolymer structure was important when the dispersions of MWCNTs were deposited as modifying layers on surfaces of SPEs, resulting in considerable increases of the electroactive surface areas and great acceleration of the electron transfer rate. The SPE/(P*n*BMA_x_-*b*-PDMAEMA_y_ + MWCNT) constructs were further exploited for direct electrochemical oxidation of the guanine (G) and adenine (A) residues in a model salmon sperm double-stranded DNA (dsDNA). Two well-defined irreversible oxidation peaks were observed at about +600 and +900 mV, corresponding to the electrochemical oxidation of G and A residues, respectively. A multi-parametric optimization of dsDNA electrochemistry enables one to get the limits of detection (LOD) as low as 5 μg/mL (0.25 μM) and 1 μg/mL (0.05 μM) for G and A residues, respectively. The achieved sensitivity of DNA assay enables quantification of the A and G residues of dsDNA in the presence of human serum and DNA in isolated human leukocytes.

## 1. Introduction

Deoxyribonucleic acid (DNA) is one of the main biomolecules; it possesses key functions in transcription, translation, and replication. The quantification of DNA molecules in biological samples is of great importance from diagnostic, prognostic, and therapeutic viewpoints [1,2,3,4,5,6]. Nucleic acids (DNA and RNA), nucleotides and oligonucleotides are recognized as markers of many pathological states. Graft DNA circulating in transplant recipients has been proposed as a potential biomarker of organ rejection or cellular graft injury [7]. Copy number of leukocyte mitochondrial DNA was already reported as a potential biomarker indicating poor outcome in biliary atresia and its association with oxidative DNA damage and telomere length [8]. The levels of purines in plasma, serum, and urine and the concentration change of adenine in DNA could be considered as indications of carcinoma or liver diseases [9,10,11,12].

Direct electroanalysis of DNA is typically based on the appearance of individual oxidative peaks of purines: guanine (G) and adenine (A) (typically at < 1.0 V potentials) and pyrimidines: cytosine (C) and thymine (T) (at higher positive potentials) [9,10,11,12,13]. It is in demand for applications in point-of-care medical diagnosis, genome research, and forensic science diagnostics of genetic and infectious diseases [14,15,16,17]. This electrochemical approach is reagentless, free from labeling (label-free), and does not require any hybridization or enzymatic reactions. There are published results on very sensitive electrochemical detection of individual heterocyclic bases; specifically, A and G. The detection limits for them can be as low as nanomolar and even subnanomolar [18]. Many works report that the developed electrochemical setups are potentially applicable for the detection of nucleotide residues in DNA on the basis of calibration curves for the individual heterocyclic bases [9,10,11,12,13,19,20]. A direct electrochemical quantification of dsDNA represents a more challenging task. It is worth noting that lower amounts of A and G residues are accessible for electrooxidation in the long rigid double-stranded DNA (dsDNA) than in short oligodinucleotides, flexible single-stranded DNA (ssDNA), or individual heterocyclic bases. Hence, an electrochemical signal coming from dsDNA is weaker [21], lowering the sensitivity of analysis [10,11,22,23].

In many cases the electroanalysis of nucleic acids and heterocyclic bases, nucleotides, and modified nucleotides is performed with screen-printed electrodes, SPEs. SPEs can be classified as sensors of the cheap and disposable variety, which are suitable for analysis of biological samples. SPEs are widely used for experiments with plasma, blood serum, whole blood, urine, saliva, tissues, cells, or exhaled condensate [24].

Carbon nanomaterials have great potential as modifiers or catalysts of different types of electrodes, which are applied for enhancing and improvement of electron transfer reactions [25,26]. Specifically, multiwalled carbon nanotubes (MWCNTs) possess unique properties that are very suitable for electroanalytical purposes, such as good conductivity, high surface area, and a high surface-to-volume ratio [27,28,29]. Moreover, a π–π electronic system of MWCNTs can interact with nucleobases and additionally facilitate electron transfer between an electrode and DNA [25,26,27,28,29]. However, MWCNTs are hardly dispersible in most conventional solvents, and that makes them not easy-to-handle. The main strategy to overcome this disadvantage is dispersing of MWCNTs in solutions of synthetic or natural polymers [23,30,31,32,33,34].

Amphiphilic ionic diblock copolymers featuring both hydrophobic and ionic blocks in one macromolecule could represent even more efficient dispersants of carbon nanomaterials. Due to their amphiphilic structure, such materials are good dispersants for carbon nanomaterials. Moreover, the charged groups provide the subsequent immobilization of analytes, while the presence of a hydrophobic segment preferably with a low or moderate glass transition temperature *T*_g_ might considerably facilitate the modification of hydrophobic carbon surfaces [23,34,35,36].

This paper demonstrates a beneficial application of amphiphilic ionic poly(*n*-butyl methacrylate)-*block*-poly(2-(dimethylamino)ethyl methacrylate)s (P*n*BMA_x_-*b*-PDMAEMA_y_) diblock copolymers for dispersing of MWCNTs in aqueous media, a subsequent modification of SPEs, and the use of the modified SPEs in DNA electrochemistry. We expect multiple functions from P*n*BMA_x_-*b*- PDMAEMA_y_: they can be used (1) as efficient dispersants for MWCNTs, (2) as binders enabling SPE/MWCNTs integrity, and (3) as host matrices, which can electrostatically bind DNA. To understand more what input could impart the structural characteristics and the physico-chemical properties of the diblock copolymer at each stage of electrochemical performance, we synthesized three different diblock copolymers, P*n*BMA_40_-*b*-PDMAEMA_40_, P*n*BMA_40_-*b*-PDMAEMA_120_, and P*n*BMA_70_-*b*-PDMAEMA_120_, which differ in molecular weight, lengths of the blocks and the block length ratio.

In this paper, we highlight the preparation of stable aqueous dispersions of MWCNTs, using P*n*BMA_x_-*b*-PDMAEMA_y_ diblock copolymers as dispersants. The following modification of SPEs by P*n*BMA_x_-*b*-PDMAEMA_y_ + MWCNT dispersions allows us to fabricate easy-preparable and universal sensor surfaces, whose analytical performance is demonstrated herein for direct electrochemical analysis of dsDNA in model solutions and in biosamples.

## 2. Materials and Methods 

Methyl α-bromoisobutyrate (≥ 99.0%), *N*-butyl-2-pyridylmethanimine (Nbpmi, 97%), 1,1,4,7,10,10-hexamethyltriethylenetetramine (HMTETA, 97%), *N,N,N′,N′′,N′′*-pentamethyl diethylenetriamine (PMDETA, 99%), CuBr (99.999%), and CuBr_2_ (99%) were obtained from Sigma-Aldrich and used as received. *n*-Butyl methacrylate (*n*BMA, 99.0%) was purchased from Sigma-Aldrich and 2-(dimethylamino)ethyl methacrylate (DMAEMA, > 98.5%) from TCI Chemicals (Tokyo, Japan) and run over an AlO_x_ column before use. MWCNTs with a mean diameter of 9.5 nm and a length of 1 µm and dsDNA from salmon sperm (*M*_w_ 10 – 30 kDa) with water content of ≤ 10% were obtained from Sigma-Aldrich. K_3_[Fe(CN)_6_] and K_4_[Fe(CN)_6_] were purchased from Reakhim (Moscow, Russia). A phosphate buffer solution of pH 7.4 containing NaCl (100 mM potassium phosphate with 50 mM NaCl of pH 7.4) was prepared by mixing stock solutions of 100 mM KH_2_PO_4_/50 mM NaCl and 100 mM K_2_HPO_4_/50 mM NaCl and further adjusting the pH-value to pH 7.4. Milli-Q water (18.2 MΩ cm) purified with a Milli-Q water purification system by Millipore was used for preparation of all aqueous solutions.

Serum samples were received from Sigma-Aldrich. The peripheral blood of donors was obtained from a blood bank and was used as a source of human leukocytes. The informed signed consent was received from each person. Leukocyte DNA was extracted from peripheral blood using the GE Healthcare DNA Mini Kit (Buckinghamshire, UK) according to the manufacturer’s protocol. The study protocol conformed to the ethical standards outlined in the Declaration of Helsinki and was approved by the Institutional Review Board of the V.N. Orekhovich Institute of Biomedical Chemistry (Moscow, Russia).

### 2.1. Synthesis of PnBMA-Br Macroinitiators

The synthesis of macroinitiators was carried out according to a procedure published in [37].

**DP of 40:***N*-Butyl methacrylate, methyl-α-bromoisobutyrate, Nbpmi, and toluene (66 *v*.%) were degassed *via* four freeze-pump-thaw cycles. Then, the mixture was added to CuBr, which was placed in a flask under argon. The reaction was carried out under argon in an oil bath at 90 °C for 75 min. The polymerization was terminated by cooling to room temperature and adding of a small amount of methanol. After removal of the Cu-catalyst through an AlO_x_ column, the product was further purified *via* dialysis against tetrahydrofuran (MWCO = 1000 g/mol).

Molar ratio of monomer/initiator/ligand/CuBr: [50]/[1]/[2]/[1].

Characterization by ^1^H Nuclear Magnetic Resonance (^1^H–NMR) Spectroscopy

^1^H–NMR (300 MHz, CDCl_3_, δ): 4.24–3.95 (m, –COO–CH_2_–), 3.62 (s, –O–CH_3_), 1.90–1.10 (m, –CH_2_–), 1.11– 0.72 (–C–CH_3_) ppm.

Characterization by Size Exclusion Chromatography (SEC)

SEC (PMMA calibration, chloroform/*iso*-propanol/triethylamine): *M*_n_ = 5600 g/mol, *M*_w_ = 8300 g/mol, *Ð* = 1.47.

**DP of 70:***N*-Butyl methacrylate, methyl-α-bromoisobutyrate, PMDETA, and anisole (50 *v*.%) were degassed *via* four freeze-pump-thaw cycles. Then, the mixture was added to CuBr and CuBr_2_, which were placed in a flask under argon. The reaction was carried out under argon in an oil bath at 60°C for 60 min. The polymerization was terminated by cooling to room temperature and adding of a small amount of methanol. After removal of the Cu-catalyst through an AlO_x_ column, the product was further purified *via* dialysis against tetrahydrofuran (MWCO = 3500 g/mol).

Molar ratio of monomer/initiator/ligand/CuBr/CuBr_2_: [200]/[1]/[0.5]/[0.5]/[0.05].

Characterization by ^1^H Nuclear Magnetic Resonance (^1^H–NMR) Spectroscopy

^1^H–NMR (300 MHz, CDCl_3_, δ): 4.24–3.95 (m, –COO–CH_2_–), 3.62 (s, –O–CH_3_), 1.90–1.10 (m, –CH_2_–), 1.11–0.72 (–C–CH_3_) ppm.

Characterization by Size Exclusion Chromatography (SEC)

SEC (PMMA calibration, chloroform/triethylamine/*iso*-propanol): *M*_n_ = 10000 g/mol, *M*_w_ = 12400 g/mol, *Ð* = 1.24.

### 2.2. Synthesis of PnBMA-b-PDMAEMA Diblock Copolymers

DMAEMA, toluene (66 *v*.%) and HMTETA were degassed *via* four freeze-pump-thaw cycles and afterwards added to P*n*BMA–Br and CuBr under argon. Then, the reaction mixture was placed in an oil bath at 90 °C and stirred under argon for a certain time. The reaction was terminated by cooling to room temperature and the addition of methanol. After removal of the Cu-catalyst through an AlO_x_ column, the solvent was removed under reduced pressure and the polymer isolated as a slightly yellow solid or the product was further purified *via* dialysis against tetrahydrofuran (MWCO = 25000 g/mol).

Molar ratio of monomer/initiator/ligand/CuBr: [100]/[1]/[1]/[1] or [150]/[1]/[1]/[1].

In case of P*n*BMA_70_-*b*-PDMAEMA_120_, CuBr_2_ was added and the molar ratio of monomer/ initiator/ligand/CuBr/CuBr_2_ changed to [150]/[1]/[1]/[1]/[0.1].

Characterization by ^1^H Nuclear Magnetic Resonance (^1^H–NMR) Spectroscopy

^1^H–NMR (300 MHz, CDCl_3_, δ): 4.18–4.01 (m, –COO–CH_2_–), 3.95–3.85 (m, –COO–CH_2_–), 3.63 (s, –O–CH_3_), 2.72–2.47 (m, –N–CH_2_–), 2.44–2.26 (m, –N–(CH_3_)_2_), 2.02 1.90–1.10 (m, –CH_2_–), 2.02–0.82 (–C–CH_2_, –CH_2_–CH_2_, –CH_2_–CH_3_) ppm.

Characterization by Size Exclusion Chromatography (SEC)

SEC (PMMA calibration, chloroform/triethylamine/*iso*-propanol): *M*_n_ = 12000 g/mol, *M*_w_ = 17200 g/mol, *Ð* = 1.43.

SEC (PMMA calibration, chloroform/triethylamine/*iso*-propanol): *M*_n_ = 15000 g/mol, *M*_w_ = 19500 g/mol, *Ð* = 1.30.

SEC (PMMA calibration, chloroform/triethylamine/*iso*-propanol): *M*_n_ = 27400 g/mol, *M*_w_ = 39700 g/mol, *Ð* = 1.45.

Exemplary ^1^H-NMR spectra of P*n*BMA_40_–Br and P*n*BMA_40_-*b*-PDMAEMA_40_ are given in Figures S1 and S2, respectively.

### 2.3. Dispersing of Carbon Nanomaterials

Aqueous solutions of P*n*BMA_x_-*b*-PDMAEMA_y_ diblock copolymers were prepared at a concentration of 5 g/L by their direct dissolution in acidified water at pH 3, at which all DMAEMA monomer units of the diblock copolymers are protonated. To disperse carbon nanomaterials, a portion of MWCNTs was added to an aqueous solution of the diblock copolymer in such a way that each 1 mL of the (P*n*BMA_x_-*b*-PDMAEMA_y_ + MWCNT) mixture contained 1.0, 1.5, 2.0, 2.5, or 3.0 mg of the carbon nanomaterial. Then, the mixtures were sonicated in Ultrasound Desintegrator SONOPULS HD 4100 (Bandelin, Germany) at 30% power in pulse regime for 30 min.

### 2.4. ^1^H Nuclear Magnetic Resonance (^1^H–NMR) Spectroscopy

^1^H-NMR measurements were performed on a Bruker AC 300 MHz using CDCl_3_ as solvent.

### 2.5. Size Exclusion Chromatography (SEC)

SEC traces were measured using a Shimadzu system equipped with a CBM-20 A system controller, a LC-10 VP pump, a RID-10A refraction index detector, and a PSS SDV guard/linear S column. As eluent, a mixture of chloroform/triethylamine/*iso*-propanol [94/2/4] (*v*/*v*/*v*) was used and measurements recorded with a flow rate of 1 ml/min and 40 °C.

### 2.6. Transmission Electron Microscopy (TEM)

For TEM measurements from aqueous solutions, copper grids were rendered hydrophilic by argon plasma cleaning for 2 min (Diener Electronics); 10 µL each of the respective sample solutions were applied onto the grid, and excess sample was blotted with filter paper. TEM images were acquired with a 200 kV FEI Tecnai G2 20 equipped with a 4k × 4k Eagle HS CCD and a 1k × 1k Olympus MegaView camera for overview images.

### 2.7. CryogenicTransmission Electron Microscopy (cryo-TEM)

Cryo-TEM measurements were performed on a FEI Tecnai G2 20 cryo-Transmission Electron Microscope (Jena Center for Soft Matter, Jena, Germany). The acceleration voltage was set to 200 kV. Samples were prepared on Quantifoil grids (3.5/1) after cleaning by argon plasma treatment for 120 s; 8.5 µL of the solutions were blotted by using a Vitrobot Mark IV. Samples were plunge-frozen in liquid ethane and stored under nitrogen before being transferred to the microscope utilizing a Gatan transfer stage. TEM images were acquired with a 200 kV FEI Tecnai G2 20 equipped with a 4k x 4k Eagle HS CCD and a 1k x 1k Olympus MegaView camera.

### 2.8. Electrochemical Measurements

Cyclic voltammetry (CV) and differential pulse voltammetry (DPV) measurements were performed using an Autolab PGSTAT12 potentiostat/galvanostat (Metrohm Autolab, the Netherlands) equipped with the GPES software, version 4.9.7 (Metrohm Autolab, the Netherlands). All electrochemical experiments were carried out at room temperature in 100 mM potassium phosphate with 50 mM NaCl of pH 7.4. CV experiments with ferricyanide were carried out in a 1 mL electrochemical cell by potential sweeping from an initial potential of −0.6 V to an end-point potential of +0.6 V at different scan rates in a range of 10 – 100 mV/s.

Three-pronged SPEs purchased from Color Electronics (http://www.colorel.ru; Moscow, Russia) were used for the electrode preparation. They consist of a round graphite working electrode (2 mm in diameter) surrounded by a graphite ringed auxiliary counter-electrode and an Ag/AgCl reference electrode. For the preparation of modified electrodes, 2 µL of the corresponding (P*n*BMA_x_-*b*-PDMAEMA_y_ + MWCNT) dispersion was dropped onto the working electrode and incubated for 15 min at 37 °C until complete drying. Then, SPE/(P*n*BMA_x_-*b*-PDMAEMA_y_ + MWCNT) were pretreated by two DPV scans in the range of +0.2 – +1.2 V in a working buffer, washed with water, and dried in the open air. Such modified electrodes were stored refrigerated at +4 °C until measurement on the same day.

For further incorporation of the analyte (dsDNA), the working area of each SPE preliminary modified by the corresponding (P*n*BMA_x_-*b*-PDMAEMA_y_ + MWCNT) dispersion was covered by a 60 μL drop of the dsDNA solution with a concentration of interest, which was prepared in 100 mM potassium phosphate with 50 mM NaCl of pH 7.4. The solution of dsDNA was incubated at room temperature for 15 or 60 min, as specifically indicated in legends to figures. Then, DPV technique was employed to follow direct electrochemical oxidation of dsDNA. A horizontal regime of measurements was used for all experiments with DNA. The following DPV parameters were used: potential range of +0.2 – +1.2 V, pulse amplitude of 0.025 V, potential step of 0.005 V, pulse duration of 50 ms, and modulation amplitude of 0.05 V. All potentials were referred to the Ag/AgCl reference electrode. When it was necessary, the obtained raw DPV data were treated using derivative calculations of the GPES software, which allowed enhancing the sensitivity by improving a signal-to-noise ratio of the DPV analysis.

Human serum was diluted for 10 times with electrolyte buffer (100 mM potassium phosphate with 50 mM NaCl of pH 7.4). The diluted serum samples were spiked with certain amounts of dsDNA solution and the resulting samples were assayed by DPV technique using modified SPEs as described above.

### 2.9. Spectrophotometric Measurements

A NanoDrop® ND-1000 UV-Vis Spectrophotometer (Thermo Fisher Scientific, USA) was used for determination of the concentration of dsDNA or DNA from human leukocytes.

## 3. Results and Discussion

### 3.1. Preparation and Characterization of (PnBMA_x_-b-PDMAEMA_y_ + MWCNT) Dispersions

Polymeric materials of amphiphilic nature can provide good dispersibility of MWCNTs in aqueous media and ensure long-term colloidal stability of such systems, as we demonstrated earlier for polymers of various structure and composition, such as imidazolium-based poly(ionic liquid)s [23], cationic poly(1,2-butadiene)-*block*-poly(2-(dimethylamino)ethyl methacrylate) diblock copolymers [34], anionic poly(*n*-butylacrylate)-*block*-poly(acrylic acid) diblock copolymers [36], and poly(dehydroalanine)-based graft copolymers [38]. The amphiphilic nature of the polymer is of key importance for successful dispersing of superhydrophobic carbon nanomaterials to individual fragments (particles) in aqueous media. While hydrophobic moieties provide disintegration to smaller fragments, the hydrophilic segments support their colloidal stability and prevent further aggregation or precipitation. Thus, the process of dispersing implies the effective interaction between the polymer and the carbon nanomaterial.

**Figure 1:**
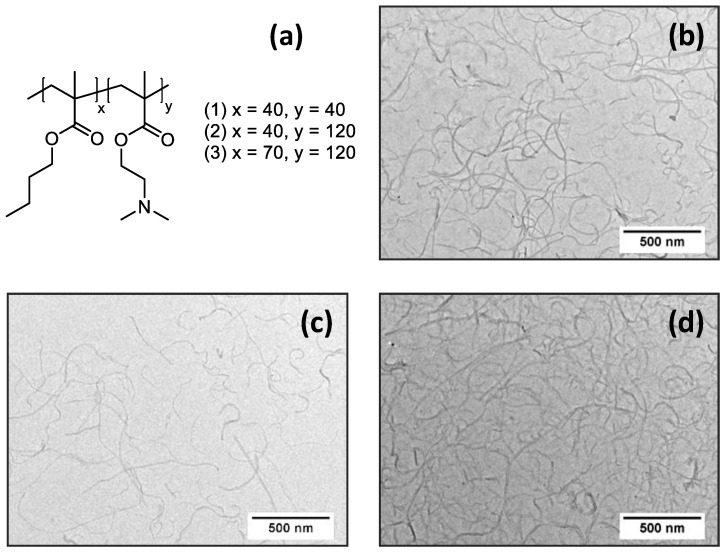
(**a**) The structure of P*n*BMA_x_-*b*-PDMAEMA_y_ diblock copolymers; (**b-d**) TEM micrographs of the (P*n*BMA_40_-*b*-PDMAEMA_40_ + MWCNT) (**b**), (P*n*BMA_40_-*b*-PDMAEMA_120_ + MWCNT) (**c**), and (P*n*BMA_70_-*b*-PDMAEMA_120_ + MWCNT) (**d**) dispersions. The (P*n*BMA_x_-*b*-PDMAEMA_y_ + MWCNT) dispersions were prepared by ultrasonication of 1 g/L of the MWCNTs in a 5 g/L aqueous solution of the corresponding diblock copolymer at pH 3.

On the other hand, the efficiency of carbon nanomaterials for electrochemistry applications depends on the effective coupling of carbon nanomaterials to an electrode surface for electron transfer. Here the polymer wrapping layer might serve as insulator, which deteriorates or even completely prevents electron exchange. Hence, the structure of the amphiphilic polymer, especially the hydrophobic-hydrophilic balance, has primary importance in the application both for the dispersing of carbon nanomaterials and for the electrochemical measurements. In case of amphiphilic diblock copolymers, this balance can be regulated by the block length ratio and the overall molecular weight. In this context, we prepared three poly(*n*-butyl methacrylate)-*block*- poly(2-(dimethylamino)ethyl methacrylate) (P*n*BMA-*b*-PDMAEMA) diblock copolymers, which differ in lengths of the blocks: P*n*BMA_40_-*b*-PDMAEMA_40_; P*n*BMA_40_-*b*-PDMAEMA_120_; and P*n*BMA_70_-*b*-PDMAEMA_120_ (Figure 1a). Here the number-average degrees of polymerization of the corresponding blocks are given as subscripts. These diblock copolymers each comprise both a hydrophobic P*n*BMA block with a moderate glass transition temperature, *T*_g_ [39], and a hydrophilic chargeable PDMAEMA segment. In aqueous media, these materials undergo self-assembly into micelles with a P*n*BMA core and a PDMAEMA corona that was confirmed by cryo-TEM. The typical appearance of the micelles is shown in Figure S3. The solution behavior of P*n*BMA_x_-*b*-PDMAEMA_y_ diblock copolymers is in good agreement with former published data for similar diblock copolymers [40].

Potentiometric titration allowed us to examine how the protonation degree α of P*n*BMA_x_-*b*- PDMAEMA_y_ diblock copolymers changes with pH in their solutions. It was shown that the diblock copolymers undergo a transition from the fully protonated (charged) state to the fully deprotonated (non-charged) state in the pH-window of 4.5 – 8.5 (Figure S4). The apparent (at α = 0.5) and characteristic p*K*_a_-values are summarized in Table S1. One should note that both the trends of the pH-dependence of α and p*K*_a_-values are very similar for different P*n*BMA_x_-*b*-PDMAEMA_y_ diblock copolymers.

Based on potentiometric titration data and our previous experience [34], we assume that due to their pronounced amphiphilic character P*n*BMA_x_-*b*-PDMAEMA_y_ diblock copolymers will easily disperse carbon nanomaterials such as MWCNTs. Furthermore, the best colloidal stability of dispersions is expected in acidic media where PDMAEMA blocks are in their charged (protonated) state. However, one cannot obtain homogeneous dispersions of MWCNTs by simple mixing of MWCNT powder with a solution of P*n*BMA_x_-*b*-PDMAEMA_y_ at pH 3 (corresponding to the fully protonated PDMAEMA blocks) [23,34,36]. Only after high energy ultrasound treatment, the disentanglement of MWCNTs bundles to individual MWCNTs was done, and we obtained very homogenous black suspensions (“inks”), which were colloidally stable at room temperature for at least several months (> 7 months).

The efficiency of dispersing was further evaluated by TEM. Typical TEM micrographs show the dominant presence of individual MWCNTs (Figure 1b,c,d) in their aqueous dispersion prepared with each of three diblock copolymers with no substantial difference between the different samples.

### 3.2. Electrochemical Characterization of Electrodes Modified by (PnBMA_x_-b-PDMAEMA_y_ + MWCNT) Dispersions

Being very easy-to-handle, the prepared dispersions of MWCNTs in aqueous solutions of P*n*BMA_x_-*b*-PDMAEMA_y_ diblock copolymers were further drop-casted as small-volume drops (2 μL) onto an active electrode area of SPEs. After drying, the modified electrodes were ready-to-use in electrochemistry. Upon a modification, the P*n*BMA_x_-*b*-PDMAEMA_y_ diblock copolymers acquired a new function—they acted as polymeric binders that can provide sufficient integrity for a deposited layer of MWCNTs on the SPE.

The electrochemical characterization of the electroactive surface area of the modified electrodes was performed by CV technique in a solution of K_3_[Fe(CN)_6_]/K_4_[Fe(CN)_6_] redox probe at different scan rates (Figures S5–S12). The values of *E*_red_, *E*_ox_, Δ*E*, *E*_1/2_, *I*_red_, and *I*_ox_ were determined at a scan rate of 50 mV/s and were compared to the naked SPE (Table 1). The data demonstrate that the naked electrode shows a pair of redox peaks with a wide peak-to-peak separation of 500 mV and low current responses, which both point to a hindered electrochemical process. Alternatively, the pair of well-defined reversible redox peaks with a narrow peak-to-peak separation of < 105 mV was found in all cases for the SPEs, which were modified by the dispersions of MWCNTs in aqueous solutions of P*n*BMA_x_-*b*-PDMAEMA_y_ diblock copolymers. This implies that more reversible redox performance of [Fe(CN)_6_]^3-/4−^ occurs when MWCNTs are used. Furthermore, a considerable increase in the current response was found as well, pointing to enhanced electron transfer properties and increased mass transfer for the SPEs, which were modified by dispersions of MWCNTs in aqueous solutions of P*n*BMA_x_-*b*-PDMAEMA_y_ diblock copolymers.

As seen from the insets of Figures S6–S12, the anodic/cathodic peak currents, *I_p_,* exhibit a linear relationship with the square root of the scan rate, *v*, in the range of 10 – 100 mV/s, confirming a chemically reversible diffusion-controlled redox reaction [41]. The electroactive surface area, *A* (cm^2^), of the electrode can be determined from the slope of the dependence of *I_p_ vs*. *ν*^1/2^ using the Randles–Sevcik equation (more details are given in the Supplementary Materials). The calculated values of *A* are also summarized in Table 1. A considerable increase in electroactive surface area was found for SPEs, which were modified by (P*n*BMA_x_-*b*-PDMAEMA_y_ + MWCNT) dispersions. The effect depends on the used diblock copolymer; the highest 11-fold increase of *A* was observed when dispersions of MWCNTs in aqueous solutions of the P*n*BMA_70_-*b*-PDMAEMA_120_ diblock copolymer were applied for modification of the SPEs. An even more pronounced 23 – 34-fold enlargement in *A* can be induced by increasing the content of MWCNTs, as demonstrated for the SPE/(P*n*BMA_40_-*b*- PDMAEMA_120_ + MWCNT_n) constructs, where n denotes the concentration of MWCNTs in their dispersion, which ranged from 1 to 3 g/L. As seen from the data presented in Table 1, the highest 34-fold increase in electroactive surface area was found for the SPE/(P*n*BMA_40_-*b*-PDMAEMA_120_ + MWCNT_2) construct with 2 g/L concentration of MWCNTs in the dispersion used for modification of the SPEs.

Thus, the obtained results convincingly demonstrate that the beneficial modification of the SPEs by the dispersions of MWCNTs in aqueous solutions of P*n*BMA_x_-*b*-PDMAEMA_y_ diblock copolymers leads to a considerable increase in electroactive surface area both due to the specific structure of the copolymer (the optimum hydrophobic-hydrophilic balance and the optimum total length of the macromolecule) and the optimum content of MWCNTs. Remarkably, the determined values of electroactive surface area strongly surpass our former results on MWCNTs dispersed in aqueous solutions of imidazolium-based poly(ionic liquid)s [23] or the PB_290_-*b*-PDMAEMA_240_ diblock copolymer [34].

### 3.3. Characterization and Quantification of Electrochemical dsDNA Assay

Apart from being polymeric binders that provide sufficient integrity of a deposited layer of MWCNTs upon electrode modification, the P*n*BMA_x_-*b*-PDMAEMA_y_ diblock copolymers themselves can also act as a host matrix. Indeed, their protonated DMAEMA moieties are expected to efficiently anchor oppositely charged target analytes (here negatively charged dsDNA), thereby providing potential advantages for electrochemical measurements.

#### 3.3.1. Optimization of Electrochemistry of dsDNA for Different Electrode Modifications

The solutions of dsDNA were prepared in 100 mM potassium phosphate with 50 mM NaCl at physiological pH value of 7.4 and were directly deposited onto the surfaces of the SPEs, which were modified by dispersions of MWCNTs in aqueous solutions of P*n*BMA_x_-*b*-PDMAEMA_y_ diblock copolymers. A short preincubation for 15 min at room temperature was already sufficient for the binding of negatively charged dsDNA to the oppositely charged PDMAEMA blocks of the copolymers present on the surface of the modified SPEs. It is worth noting that the degree of protonation of the PDMAEMA block of 25 – 30% at physiological pH of 7.4 (see potentiometric titration curves given in the Figure S4) was sufficient for the strong multipoint anchoring of DNA. This corresponds to the results we already reported for the pH-dependent interaction of similar diblock copolymers deposited onto solid surfaces with enzymes and proteins [35,40,42].

**Figure 2:**
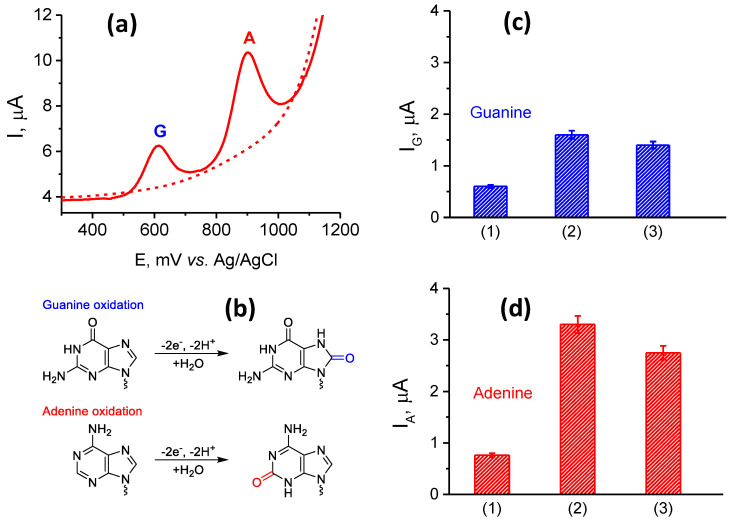
(**a**) Typical DPVs for the SPE/(P*n*BMA_40_-*b*-PDMAEMA_120_ + MWCNT_1) in the absence (dashed line) or in the presence (solid line) of dsDNA. (**b**) The mechanisms of electrochemical oxidation of guanine and adenine residues. (**c**, **d**) Comparative current responses for guanine (**c**) or adenine (**d**) peaks for (1) SPE/(P*n*BMA_40_-*b*-PDMAEMA_40_ + MWCNT_1)/dsDNA, (2) SPE/ (P*n*BMA_40_-*b*-PDMAEMA_120_ + MWCNT_1)/dsDNA, and (3) SPE/(P*n*BMA_70_-*b*-PDMAEMA_120_ + MWCNT_1)/dsDNA constructs. Conditions: dsDNA was deposited onto the SPE/(P*n*BMA_x_-*b*- PDMAEMA_y_ + MWCNT_1) constructs from a 1500 µg/ml solution. The incubation time after dsDNA deposition was 15 min.

DPV scanning of SPE/(P*n*BMA_x_-*b*-PDMAEMA_y_ + MWCNT)/dsDNA constructs allows one to follow direct electrochemical oxidation of dsDNA. As can be seen in Figure 2a, two clear non-overlapping peaks were observed at about +600 and +900 mV, corresponding to the electrochemical oxidations of G and A residues, respectively. The observed electrooxidation potentials were similar to the values reported elsewhere [21,34,43,44,45,46]. At the same time, no peaks were found during the potential scan in the absence of dsDNA, that is, for a reference SPE/(P*n*BMA_x_-*b*-PDMAEMA_y_ + MWCNT) construct. The known mechanism of electrochemical transformation of DNA involves the irreversible oxidation of G to 8-oxoguanine, while A is converted to 2-oxoadenine according to the scheme shown in Figure 2b [21,43,44,47,48,49].

The DPV responses of SPE/(P*n*BMA_x_-*b*-PDMAEMA_y_ + MWCNT)/dsDNA constructs obtained for 1500 µg/mL dsDNA solution used for deposition were determined after sample preincubation for 15 min. The current responses for the electrochemical oxidation of G and A residues were measured and compared with a naked SPE and SPEs modified by 1 g/L dispersions of MWCNTs in aqueous solutions of each of the three diblock copolymers: (P*n*BMA_40_-*b*-PDMAEMA_40_ + MWCNT_1), (P*n*BMA_40_-*b*-PDMAEMA_120_ + MWCNT_1), and SPE/(P*n*BMA_70_-*b*-PDMAEMA_120_ + MWCNT_1) (Figure 1b,c,d, Table 2). As one can see, the naked SPE shows one rather weak peak at about +935 mV, which corresponds to oxidation of A residues with the current responses in the range of several nA (Table 2). Alternatively, a remarkable increase in the DPV response was found for all SPE/(P*n*BMA_x_-*b*-PDMAEMA_y_ + MWCNT)/dsDNA constructs. We assume that the increase of the current responses was achieved due to an efficient dispersing of MWCNTs, which is provided by the amphiphilic nature of the diblock copolymers used. MWCNTs themselves have high specific surface area and facilitate an electron exchange between the electrode surface and nucleobases to be oxidized. Another reason is an efficient anchoring of the negatively charged DNA to the positively charged PDMAEMA blocks of the copolymers, which might increase the amount of dsDNA on the electrode surface. A comparison of the data presented in Figure 2c,d and Table 2 shows that the highest oxidative peaks were found for the SPE/(P*n*BMA_40_-*b*-PDMAEMA_120_ + MWCNT)/dsDNA construct. This finding suggests that the P*n*BMA_40_-*b*-PDMAEMA_120_ diblock copolymer possesses the optimum lengths of the hydrophobic and ionic blocks and a balance (ratio) between them to provide necessary features: good dispersibility of MWCNTs, integrity of a modifying layer, its conductivity, etc. Thus, we used the SPE/(P*n*BMA_40_-*b*-PDMAEMA_120_ + MWCNT) construct for further optimization of electrochemical experiments.

It is interesting to mention that for the SPE/(P*n*BMA_40_-*b*-PDMAEMA_120_ + MWCNT_1)/dsDNA and SPE/(P*n*BMA_70_-*b*-PDMAEMA_120_ + MWCNT_1)/dsDNA constructs, the oxidation potentials (peak positions) for electrooxidation of both G and A residues decreased for 30 – 40 mV. This finding strongly suggests facilitation of electron transfer properties for these electrode modifications, although the reason for that phenomenon is not clear yet.

**Figure 3:**
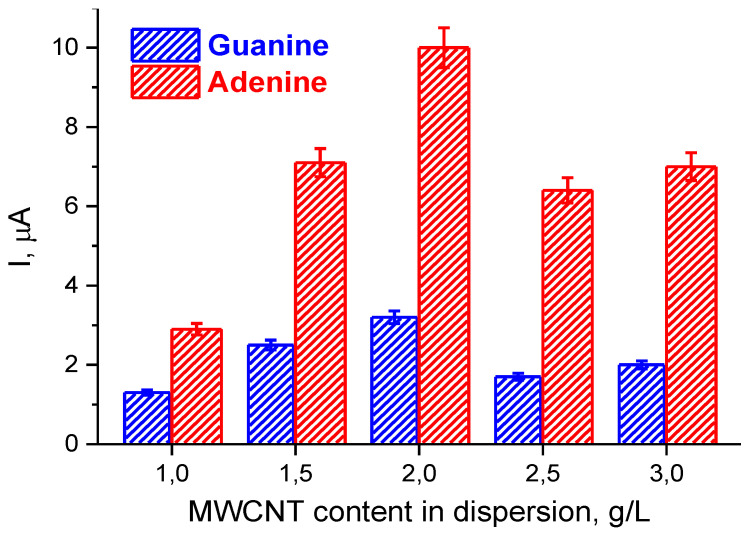
The current intensities of the DPV peaks for electrooxidation of the guanine (blue) and adenine (red) residues of dsDNA for different concentrations of MWCNTs in the (P*n*BMA_40_-*b*- PDMAEMA_120_ + MWCNT) dispersions. Conditions: dsDNA was deposited onto the SPE/(P*n*BMA_40_- *b*-PDMAEMA_120_ + MWCNT_n) constructs from a 1500 µg/ml solution. The incubation time after dsDNA deposition was 15 min.

Figure 3 shows the dependence of the current intensities of the DPV peaks for the electrooxidation of G and A residues on the concentration of MWCNTs in the dispersion used for modification of SPEs. The electrode modifications are denoted as SPE/(P*n*BMA_40_-*b*-PDMAEMA_120_ + MWCNT_n)/dsDNA. Generally, the dependences in Figure 3 demonstrate a similar trend; that is, the effect of the content of MWCNTs in their dispersions on the electroactive surface area (Table 1). The highest DPV response was found for electrooxidation of both G and A residues for the SPE/(P*n*BMA_40_-*b*-PDMAEMA_120_ + MWCNT_2)/dsDNA constructs. A concentration of MWCNTs of 2g/L appears to be optimal, which provides a compromise between colloidal stability of the (P*n*BMA_40_-*b*-PDMAEMA_120_ + MWCNT) dispersion (at a given composition and concentration of the diblock copolymer) and the efficiency of electron transfer processes in the fabricated SPE/ (P*n*BMA_40_-*b*-PDMAEMA_120_ + MWCNT)/dsDNA construct. As a result of optimization, the best SPE/(P*n*BMA_40_-*b*-PDMAEMA_120_ + MWCNT_2)/dsDNA modification was found and used for all further experiments.

#### 3.3.2. Electroanalysis of dsDNA in the Presence of Human Serum

**Figure 4:**
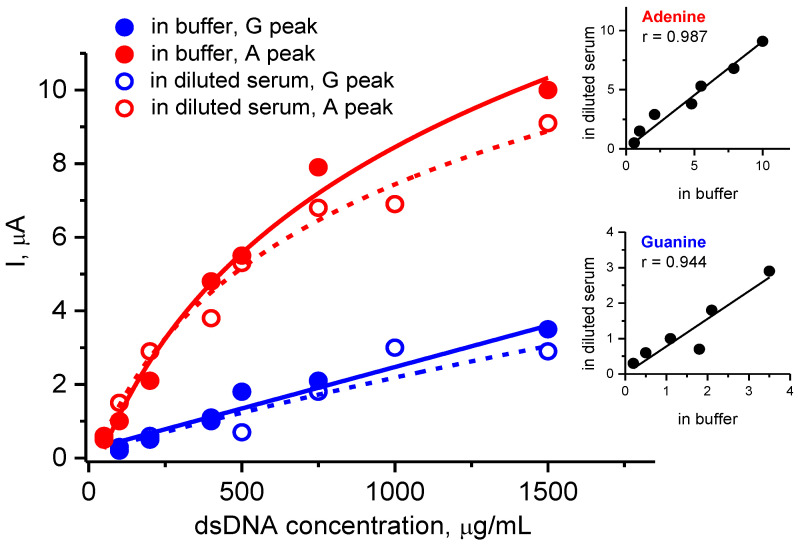
The dependences of the current intensities of the DPV peaks for electrooxidation of the guanine (blue) and adenine (red) residues on the concentration of dsDNA in a buffer media (open symbols) or dsDNA spiked into diluted human serum (closed symbols). Conditions: measurements were carried out with the SPE/(P*n*BMA_40_-*b*-PDMAEMA_120_ + MWCNT_2) construct; time of DNA incubation was 15 min; human serum was firstly diluted 10 times with electrolyte buffer (100 mM potassium phosphate with 50 mM NaCl, pH 7.4) then was spiked with a 50 – 1500 µg/mL dsDNA solution. Insets: correlations between the DPV responses of the guanine and adenine residues measured in pure buffer and in the presence of human serum.

To show analytical potential of SPE/(P*n*BMA_40_-*b*-PDMAEMA_120_ + MWCNT_2) constructs for quantitative analysis of dsDNA, we examined the dependence of DPV responses on the dsDNA concentration in the samples. Additionally, the same experiment was carried out in the presence of 10-fold diluted human serum to examine a possible interfering effect of a complex biological medium. Figure 4 demonstrates the concentration-dependent current intensities for oxidation of G and A residues of dsDNA in pure buffer and in the presence of diluted serum. The G response is a linear function of the dsDNA concentration in the whole 50 – 1500 µg/mL range examined. At the same time, the linearity for the A response was found only within 50 – 200 µg/mL range of the dsDNA concentration. Very similar concentration dependences with the same linear ranges were found in the presence of 10-fold diluted serum, although the absolute values of DPV responses were somewhat below. Nevertheless, the measured DPV responses for G and A residues of dsDNA in the buffer highly correlated with those in diluted serum (see Figure 4, Insets). Hence, the SPE/ (P*n*BMA_40_-*b*-PDMAEMA_120_ + MWCNT_2) construct appears to be potentially suitable for the quantification of dsDNA even in the presence of interfering components of biosamples.

#### 3.3.3. Electroanalysis of dsDNA at Low Concentrations

**Figure 5:**
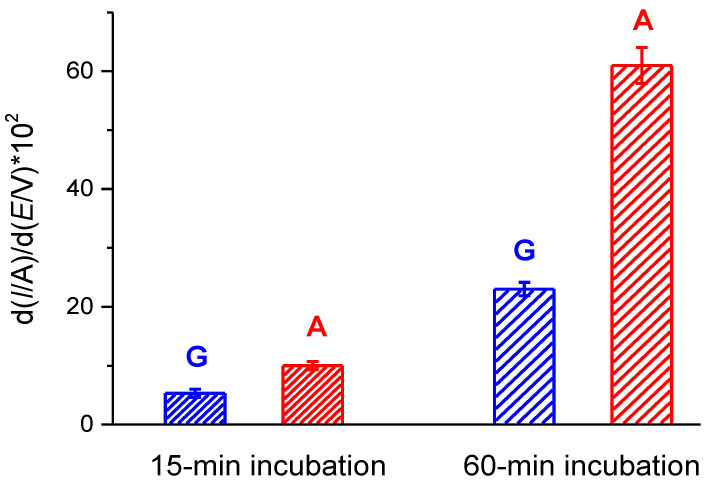
The current intensities of the differentiated DPV peaks for electrooxidation of the guanine (blue) and adenine (red) residues after incubation of a 30 μg/ml dsDNA solution on the SPE/ (P*n*BMA_40_-*b*-PDMAEMA_120_ + MWCNT_2) construct for 15 or 60 min.

The local pre-concentration of dsDNA at the electrode surface could enhance the sensitivity of the DNA assay. Knowing that, we optimized the time of incubation of dsDNA on the surface of the modified SPEs prior to the electrochemical measurement. Additionally, for a better visualization of the DPV responses, which could improve a signal-to-noise ratio, we used a known algorithm, which applies a first derivation of *I vs*. *E* curves. As can be seen from Figure 5, the increase of the preincubation time from 15 to 60 min considerably enhanced the DPV peak intensity of the G and A responses of a sample with the concentration of dsDNA of 30 μg/ml. It is also worth noting that the increased intensities of the DPV peaks were obtained due to differentiation of the raw DPV data.

**Figure 6:**
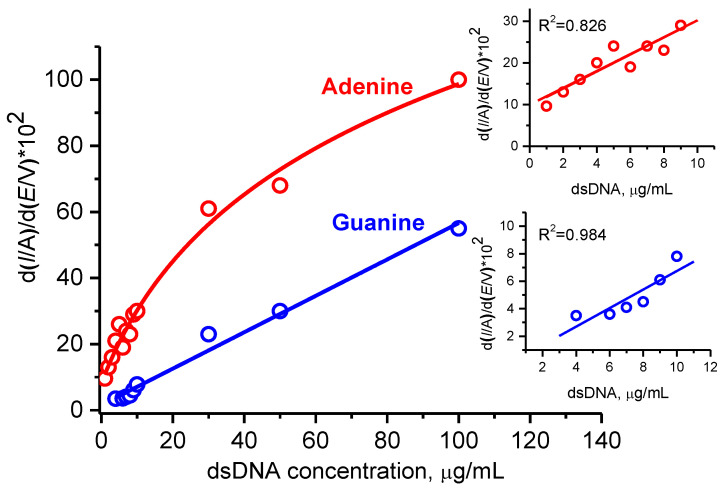
The dependences of the intensities of the differentiated DPV peaks for electrooxidation of the guanine (blue) and adenine (red) residues for the different concentrations of dsDNA. Conditions: measurements were carried out with the SPE/(P*n*BMA_40_-*b*-PDMAEMA_120_ + MWCNT_2) construct; the incubation time after dsDNA deposition was 60 min. First derivatives of the DPV raw data were used for calculating the given intensity values.

Under the aforementioned conditions of the DPV response amplification, we obtained the dependences of the intensities of the differentiated DPV peaks on the concentration of dsDNA, which varied in the range of 1 – 100 µg/mL, for the SPE/(P*n*BMA_40_-*b*-PDMAEMA_120_ + MWCNT_2) construct. The calibration curves for the G and A responses are shown in Figure 6, with the initial linear parts of the calibration curves being given in the corresponding insets. As one can see, a remarkable shift to lower concentrations of DNA was achieved (*cf*. Figure 4 and Figure 6). A linear behavior for the G response was observed in the whole 5 – 100 µg/mL range of the dsDNA concentration, while the dependence of the A response retains linearity only for the dsDNA concentrations below 10 µg/mL. The limits of detection (LOD) can be assessed as 5 μg/mL and 1 μg/mL for G and A residues, respectively. Taking into account the mean molar mass of dsDNA of salmon sperm as 20 kDa, we convert the LOD values into 0.25 μM and 0.05 μM for G and A, respectively. The obtained analytical performance of the dsDNA assay for the SPE/(P*n*BMA_40_-*b*- PDMAEMA_120_ + MWCNT_2) construct was comparable to our previously published results obtained with imidazolium-based poly(ionic liquid)s [28]. We also compared the data on sensitivity of the direct electrochemical analysis of DNA from different sources (Table S2). The comparison of our results with the LODs published by others clearly demonstrates the competitive performance of our sensing system.

#### 3.3.4. Electroanalysis of Leukocyte DNA

Leukocyte DNA has been recognized to be of therapeutic relevance [8,50,51,52], including evaluation of oxidative DNA damage or an assessment of leukocyte DNA integrity. In particular, leukocyte mitochondrial DNA is a potential biomarker in biliary atresia associated with oxidative DNA damage [8]. In Section 3.3.3., we already demonstrated the suitability of the SPE/(P*n*BMA_40_-*b*-PDMAEMA_120_ + MWCNT_2) construct for the sensitive quantification of low concentrations of model dsDNA from salmon sperm. The published values allow us to assume that human leukocyte DNA might also be assayed electrochemically with the developed and optimized SPE/(P*n*BMA_40_-*b*-PDMAEMA_120_ + MWCNT_2) construct. Here we specifically note that one should not directly compare the data on electrooxidation of A and G residues for DNA obtained from the different sources.

**Figure 7:**
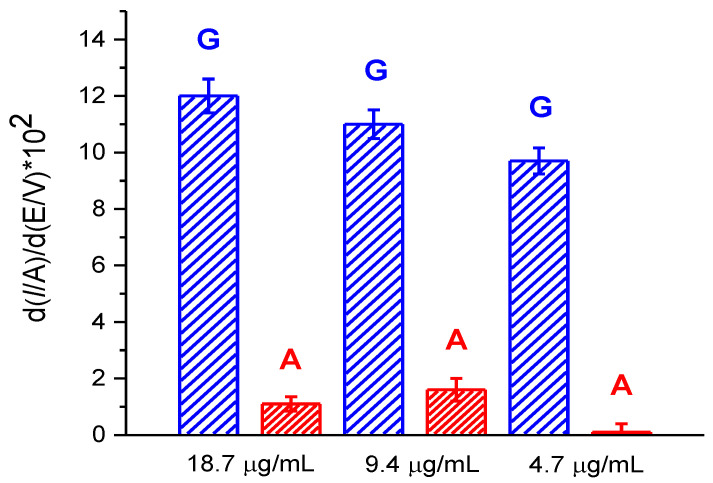
The current intensities of the differentiated DPV peaks for electrooxidation of the guanine (blue) and adenine (red) residues for 18.7, 9.35, and 4.7 μg/mL leukocyte DNA solutions assayed with the SPE/(P*n*BMA_40_-*b*-PDMAEMA_120_ + MWCNT_2) construct. Incubation time after DNA deposition was 60 min. First derivatives of the DPV raw data were used for calculating the given intensity values.

Hence, we assayed DNA isolated from human blood leukocytes to demonstrate the analytical prospects of the newly developed SPE/(P*n*BMA_40_-*b*-PDMAEMA_120_ + MWCNT_2) construct. The concentration of the isolated leukocyte DNA was determined by spectrophotometry as 186.6 μg/mL. For electrochemical measurements, the stock leukocyte DNA solution was diluted for 10, 20 or 40-times with the electrolyte buffer. Then, the samples were incubated at the electrode surface for 60 min and two non-overlapping electrochemical oxidative peaks of G and A residues at +0.620 V and +0.920 V were registered by the DPV technique for 18.7, 9.35, and 4.7 μg/ml leukocyte DNA solutions (Figure S13). The current intensities of the DPV peaks given in the Figure 7 satisfactorily correlate with sample dilution. Both G and A residues were relevantly assayed in the samples of the 18.7 and 9.35 μg/ml leukocyte DNA solutions. At the same time, the sensitivity of the SPE/(P*n*BMA_40_-*b*- PDMAEMA_120_ + MWCNT_2) construct is insufficient to observe the peak of A for more diluted sample of a 4.7 μg/mL leukocyte DNA solution.

Thus, we demonstrated that the SPE/(P*n*BMA_40_-*b*-PDMAEMA_120_ + MWCNT_2) constructs are also suitable for the assay of human leukocyte DNA at low concentrations. We suppose that such guanine/adenine (G/A) DPV peak ratio might be unique for each individual. Hence, the G/A signature (profile) of leukocyte DNA can be used for detection, quantification, and comparative analysis of DNA from different biosamples.

## 4. Conclusions

This paper highlights a beneficial application of amphiphilic ionic (P*n*BMA_40_-*b*-PDMAEMA_40_, P*n*BMA_40_-*b*-PDMAEMA_120_, P*n*BMA_70_-*b*-PDMAEMA_120_) diblock copolymers for dispersing MWCNTs in aqueous media, a subsequent efficient modification of SPEs, and the application of the modified SPEs for DNA electrochemistry.

Long-term stable and fine aqueous dispersions of MWCNTs were obtained with the use of the P*n*BMA_x_-*b*-PDMAEMA_y_ diblock copolymers, regardless of the block copolymer structure and the amount of MWCNTs used for preparing the dispersions. The effect of diblock copolymer structure is important when the dispersions of MWCNTs were deposited to build up a modifying layer of the carbon nanomaterial on a surface of the SPEs. Such modification considerably increases the electroactive surface area and improves the electron transfer rate. The highest combined effect was found for the SPE/(P*n*BMA_40_-*b*-PDMAEMA_120_ + MWCNT) construct, which was fabricated using the dispersion MWCNTs with the concentration of 2 g/L.

The SPE/(P*n*BMA_x_-*b*-PDMAEMA_y_ + MWCNT) constructs were further exploited for examining the direct electrooxidation of G and A residues in salmon sperm dsDNA used as a model nucleic acid. Two well-defined irreversible oxidation peaks corresponding to such residues were taken for the quantitative detection of dsDNA after its direct deposition onto the surface of the modified SPEs. A multi-parametric optimization of dsDNA electrochemistry (the composition of the diblock copolymer used, the content of MWCNTs, and the time of dsDNA incubation after its deposition) enables quantification of the A and G residues of dsDNA in the presence of human serum and DNA in isolated human leukocytes.

As medicine, pharmacology, biodefense, environmental studies, and agriculture urgently need sensitive, specific, and fast analytical platforms for the detection of DNA in biosamples [52,53,54], we are sure that our strategy allows one to fabricate sensing systems with favorable electrochemical activity which can be readily applied for the development of convenient and robust biosensors for numerous practical applications.

## Figures and Tables

**Table 1:** Comparison of the electrochemical characteristics of the modified screen-printed electrodes (SPEs) ^1^.

Modification Code	MWCNT Content, g/L	*E*_red_, mV	*E*_ox_, mV	Δ*E*, mV	*E*_1/2_, mV	*I*_red_, μμ	*I*_ox_, μμ	Electroactive Surface Area, *A*, cm^2^	Electroactive Surface Area Relative to the Reference Electrode (naked SPE)
Naked SPE	-	−87	421	499	167	6	5	0.0024 (8% ^2^)	1
SPE/(P*n*BMA_40_-*b*-PDMAEMA_40_+MWCNT_1)	1.0	130	235	105	183	44	36	0.012 (37%)	5
SPE/(P*n*BMA_40_-*b*-PDMAEMA_120_+MWCNT_1)	1.0	141	240	100	190	38	30	0.014 (45%)	6
SPE/(P*n*BMA_70_-*b*-PDMAEMA_120_+MWCNT_1)	1.0	125	220	95	173	104	69	0.027 (85%)	11
SPE/(P*n*BMA_40_-*b*-PDMAEMA_120_+MWCNT_1)	1.0	141	240	100	190	38	30	0.014 (45%)	6
SPE/(P*n*BMA_40_-*b*-PDMAEMA_120_+MWCNT_1.5)	1.5	125	160	35	143	197	96	0.069 (220%)	29
SPE/(P*n*BMA_40_-*b*-PDMAEMA_120_+MWCNT_2)	2.0	125	160	35	143	247	103	0.081 (259%)	34
SPE/(P*n*BMA_40_-*b*-PDMAEMA_120_+MWCNT_2.5)	2.5	125	150	25	138	227	87	0.054 (173%)	23
SPE/(P*n*BMA_40_-*b*-PDMAEMA_120_+MWCNT_3)	3.0	120	150	30	135	254	106	0.076 (241%)	32

^1^ The measurements were carried out in 5mM of K_3_[Fe(CN)_6_] at ambient temperature in potential range from −600 to +600 mV (*vs.* Ag/AgCl) for a potential scan rate of 50 mV/s. All potentials are given *vs.* Ag/AgCl reference electrode. ^2^ Percentages were calculated with respect to the geometric electrode area of 0.0314 cm^2^.

**Table 2:** Comparative electrochemical characteristics of dsDNA electrooxidation using SPE/(P*n*BMA_x_-*b*-PDMAEMA_y_ + MWCNT_1)/dsDNA constructs.

Modification	Guanine (G) Residue		Adenine (A) Residue	
	*V*, mV	*I*, µA	*V*, mV	*I*, µA
Naked SPE	-	-	935 ± 17	0.005 ± 0.0003
SPE/(P*n*BMA_40_-*b*-PDMAEMA_40_+MWCNT_1)/dsDNA	644 ± 20	0.6 ± 0.1	947 ± 40	0.6 ± 0.2
SPE/(P*n*BMA_40_-*b*-PDMAEMA_120_+MWCNT_1)/dsDNA	610 ± 10	1.5 ± 0.1	898 ± 40	3.0 ± 0.3
SPE/(P*n*BMA_70_-*b*-PDMAEMA_120_+MWCNT_1)/dsDNA	610 ± 10	1.4 ± 0.2	898 ± 40	2.4 ± 0.3

## Supplementary Materials

The following are available online at https://www.mdpi.com/2073-4360/12/7/1514/s1, Figure S1–S2: ^1^H-NMR spectra of P*n*BMA_40_-Br and P*n*BMA_40_-*b*-PDMAEMA_40_. Figure S3: The cryo-TEM micrograph of P*n*BMA_40_-*b*-PDMAEMA_40_. Figure S4: Potentiometric titration curves for PnBMA_x_-*b*-PDMAEMA_y_. Table S1: p*K*_a_ values for P*n*BMA_x_-*b*-PDMAEMA_y_. Figures S5–S12: The electrochemical characterization of the electroactive surface area of the modified SPEs. Table S2: Comparison of different sensing systems for the direct electrochemical assay of DNA. Figure S13: The appearance of the typical differentiated DPV responses for the leucocytes DNA.
