# Supplementary material for: Rational Design of Amphiphilic Diblock Copolymer/MWCNT Surface Modifiers and Their Application for Direct Electrochemical Sensing of DNA
Source: Polymers (Basel). 2020 Jul 8;12(7):1514. doi: 10.3390/polym12071514 (PMC7407114; doi:10.3390/polym12071514)
Supplement: Supplementary file 1 [file polymers-12-01514-s001.zip › polymers-836909_supplementary materials.docx]

Rational design of amphiphilic diblock copolymer/MWCNT surface modifiers and their application for direct electrochemical sensing of DNA

Larisa V. Sigolaeva ^1,2^*, Tatiana V. Bulko^1,2^, Apollinariya Yu. Konyakhina^1^, Alexey V. Kuzikov^1,2,3^, Rami A. Masamrekh^1,2,3^, Johannes B. Max^4^, Moritz Köhler^4^, Felix H. Schacher^4,5,6^, Dmitry V. Pergushov^1^, Viсtoria V. Shumyantseva^1,2,3^

^1^ Department of Chemistry, M.V. Lomonosov Moscow State University, Leninskie Gory 1/3,
119991 Moscow, Russia; lsigolaeva@belozersky.msu.ru (L.V.S.); konyakhina@list.ru (A.Yu. K.); pergush@belozersky.msu.ru (D.V.P)

^2^ V.N. Orekhovich Institute of Biomedical Chemistry, 119121 Moscow, Russia; tanyabulko@mail.ru (T.V.B.); alexeykuzikov@gmail.com (A.V.K.); rami.masamreh@yandex.ru (R.A.M.); Viktoria.Shumyantseva@ibmc.msk.ru (V.V.S.)

^3^ Pirogov Russian National Research Medical University, Ostrovitianov Street, 1, Moscow 117997, Russia

^4^ Institute of Organic Chemistry and Macromolecular Chemistry (IOMC), Friedrich-Schiller-University Jena, D-07743 Jena, Germany; johannes.max@uni-jena.de (J.M.); moritz.koehler@uni-jena.de (M.K.); felix.shacher@uni-jena.de (F.H.S.)

^5^ Jena Center for Soft Matter (JCSM), Friedrich-Schiller-University Jena, D-07743 Jena, Germany

^6^ Center for Energy and Environmental Chemistry (CEEC), Friedrich-Schiller-University Jena, D-07743 Jena, Germany

***** Correspondence: lsigolaeva@belozersky.msu.ru; Tel.: +7-495-939-40-42

**Figure S1**. ^1^H-NMR (300 MHz) spectrum of P*n*BMA_40_-Br in CDCl_3_.

**Figure S2**. ^1^H-NMR (300 MHz) spectrum of P*n*BMA_40_-*b*-PDMAEMA_40_ in CDCl_3_.

**Figure S3**: Cryo-TEM micrographs of P*n*BMA_40_-*b*-PDMAEMA_40_ in water. Micelles of P*n*BMA_40_-*b*-PDMAEMA_40_ are shown by arrows.

**Figure S4.** pH-dependences of the protonation degree α of the P*n*BMA_x_-*b*-PDMAEMA_y_ diblock copolymers. The copolymer concentration was 5 mM (in terms of the concentration of ionic groups of the PDMAEMA blocks). The titration was started from initial pH of aqueous solution of the corresponding diblock copolymer and was performed by stepwise addition of 10 µL of 10 mM HCl.

**Table S1.** Characteristic pK_a_^char.^ and apparent pK_a_^app.^ values of P*n*BMA_x_-*b*-PDMAEMA_y_.

| Diblock copolymer | pK_a_^char.^(α=0) | pK_a_^app.^(α=0.5) |
| --- | --- | --- |
| P*n*BMA_40_-*b*-PDMAEMA_40_ | 6.69 | 6.41 |
| P*n*BMA_40_-*b*-PDMAEMA_120_ | 6.85 | 6.82 |
| P*n*BMA_70_-*b*-PDMAEMA_120_ | 7.00 | 6.54 |

**Calculation of the electroactive surface area**

The electroactive surface area, *A* (cm^2^), of the electrode can be defined using the Randles–Sevcik equation:

$Ip={2.69*10}^{5}*A*\sqrt{D}* \sqrt{v}*C*n^{3/2}$ (1),

where *I_p_* is the current maximum in amps, *A* is the electroactive surface area in cm^2^, *D* is the diffusion coefficient in cm^2^/s, *ν* is the scan rate in V/s, *C* is the concentration in mol/cm^3^, and *n* is the number of electrons transferred in the redox event.

By substituting the known values of *D* = 7.6 × 10^−6^cm^2^/s, *n* = 1, and *C* = 0.005 M for the used K_3_[Fe(CN)_6_]/K_4_[Fe(CN)_6_] redox probe into the equation (1), the surface areas of each electrode modification can easily be extracted from the slope of the dependence of *I_p_* *vs.* *ν*^1/2^.

**Figure S5.** Typical CVs for the naked SPE. The measurements were carried out in 5 mM of K_3_[Fe(CN)_6_] at ambient temperature in a potential range from +700 mV to -700 mV (*vs*. Ag/AgCl) at scan rates in a range of 10–100 mV/s. Inset: The dependence of the peak current *I*_p_ on the square root of the scan rate in a range of 10–100 mV/s.

**Figure S6.** Typical CVs for the SPE/(P*n*BMA_40_-*b*-PDMAEMA_40_ + MWCNT_1) construct. The measurements were carried out in 5 mM of K_3_[Fe(CN)_6_] at ambient temperature in a potential range from +600 mV to -600 mV (*vs*. Ag/AgCl) at scan rates in a range of 10–100 mV/s. Inset: The dependence of the peak current *I*_p_ on the square root of the scan rate in a range of 10–100 mV/s.

**Figure S7.** Typical CVs for the SPE/(P*n*BMA_40_-*b*-PDMAEMA_120_ + MWCNT_1) construct. The measurements were carried out in 5 mM of K_3_[Fe(CN)_6_] at ambient temperature in a potential range from +600 mV to -600 mV (*vs*. Ag/AgCl) at scan rates in a range of 10–100 mV/s. Inset: The dependence of the peak current *I*_p_ on the square root of the scan rate in a range of 10–100 mV/s.

**Figure S8.** Typical CVs for the SPE/(P*n*BMA_70_-*b*-PDMAEMA_120_ + MWCNT_1) construct. The measurements were carried out in 5 mM of K_3_[Fe(CN)_6_] at ambient temperature in a potential range from +600 mV to -600 mV (*vs*. Ag/AgCl) at scan rates in a range of 10–100 mV/s. Inset: The dependence of the peak current *I*_p_ on the square root of the scan rate in a range of 10–100 mV/s.

**Figure S9.** Typical CVs for the SPE/(P*n*BMA_40_-*b*-PDMAEMA_120_ + MWCNT_1.5). The measurements were carried out in 5 mM of K_3_[Fe(CN)_6_] at ambient temperature in a potential range from +600 mV to -600 mV (*vs*. Ag/AgCl) at scan rates in a range of 10–100 mV/s. Inset: The dependence of the peak current *I*_p_ on the square root of the scan rate in a range of 10–100 mV/s.

**Figure S10.** Typical CVs for the SPE/(P*n*BMA_40_-*b*-PDMAEMA_120_ + MWCNT_2). The measurements were carried out in 5 mM of K_3_[Fe(CN)_6_] at ambient temperature in a potential range from +600 mV to -600 mV (*vs*. Ag/AgCl) at scan rates in a range of 10–100 mV/s. Inset: The dependence of the peak current *I*_p_ on the square root of the scan rate in a range of 10–100 mV/s.

**Figure S11.** Typical CVs for the SPE/(P*n*BMA_40_-*b*-PDMAEMA_120_ + MWCNT_2.5). The measurements were carried out in 5 mM of K_3_[Fe(CN)_6_] at ambient temperature in a potential range from +600 mV to -600 mV (*vs*. Ag/AgCl) at scan rates in a range of 10–100 mV/s. Inset: The dependence of the peak current *I*_p_ on the square root of the scan rate in a range of 10–100 mV/s.

**Figure S12.** Typical CVs for the SPE/(P*n*BMA_40_-*b*-PDMAEMA_120_ + MWCNT_3). The measurements were carried out in 5 mM of K_3_[Fe(CN)_6_] at ambient temperature in a potential range from +600 mV to -600 mV (*vs*. Ag/AgCl) at scan rates in a range of 10–100 mV/s. Inset: The dependence of the peak current *I*_p_ on the square root of the scan rate in a range of 10–100 mV/s.

Table S2. Comparison of different electrochemical sensors for the

direct electrochemical determination of DNA.

| Electrode | DNA source | Linear range | LOD | Reference |
| --- | --- | --- | --- | --- |
| OMC-CILE | Herring sperm dsDNA | 10.0 to 600.0 µg/mL  (for both A and G) | 1.2 µg/mL | [10] |
| SPGE | Intact oligodeoxynucleotide d(G_3_A_3_)_5_ | 0.022 - 0.50 µM (A) | - | [11] |
| (DomP)-s-SWNTs/GCE | Denatured fish sperm DNA | 10-600 μg/mL (A) | - | [22] |
| SPE/PIL-But@MWCNT | Salmon sperm dsDNA | 5–500 µg/mL (G)  0.5–50 µg/mL (A) | 5 µg/mL (G)  0.5 µg/mL (A) | [23] |
| SPE/(P*n*BMA_40_-*b*-PDMAEMA_120_+MWCNT) | Salmon sperm dsDNA | 5–200 µg/mL (G)  1–200 µg/mL (A) | 5 µg/mL (G)  1 µg/mL (A)^*)^ | This work |

Abbreviations: SPGE - screen printed graphite electrode; GCE – glassy carbon electrode; (DomP)-s-SWNTs – semiconducting single walled carbon nanotubes modified by 1-docosyloxylmethylpyrene; OME - ordered mesoporous carbon; CILE - carbon ionic liquid electrode (carbon electrode modified by ionic liquid 1-ethyl-3-methylimidazolium ethylsulphate); PIL-But - poly(1-butyl-3-vinylimidazolium bromide).

^*)^ Taking into account the mean molar mass of dsDNA of salmon sperm as 20 kDa, we convert the LOD values into 0.25 μM and 0.05 μM for G- and A residues, respectively.

**A**

**B**

**C**

**Figure S13.** Appearance of the typical differentiated DPVs responses for the 18.7 μg/ml (A), 9.35 μg/ml (B), and 4.7 μg/ml (C) leukocyte DNA solutions measured for the SPE/(P*n*BMA_40_-*b*-PDMAEMA_120_ + MWCNT_2) electrode modification after 60 min sample incubation after DNA deposition.
